# Supplementary material for: Proteasomal degradation of BRAHMA promotes Boron tolerance in Arabidopsis
Source: Nat Commun. 2018 Dec 11;9:5285. doi: 10.1038/s41467-018-07393-6 (PMC6290004; doi:10.1038/s41467-018-07393-6)
Supplement: Supplementary file 1 — Supplementary Information [file 41467_2018_7393_MOESM1_ESM.pdf]

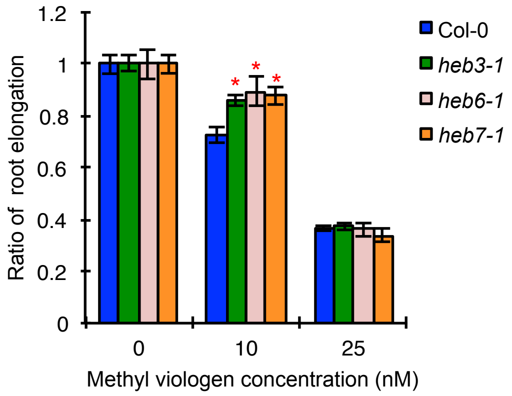

Supplementary Figure 1: Sensitivity of root growth of Col-0 and the *heb* mutants to oxidative stress

Oxidative stress was caused by methyl viologen. After 5 d incubation on normal media, plants were transferred to media containing indicated concentrations of methyl viologen. Then the elongated primary root length during additional 4 d incubation was measured. Values were represented as ratio relative to the value under 0 nM condition (n=13−16, average ± s.e.m; **P*<0.05, compared with Col-0, Student's *t*-test).

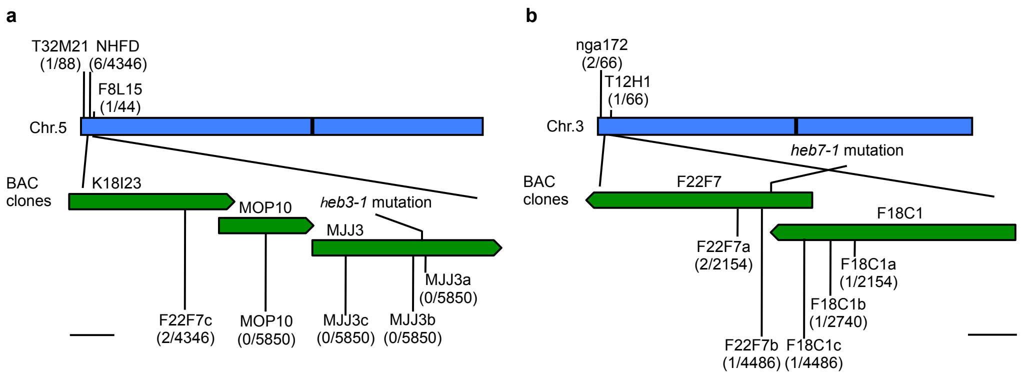

Supplementary Figure 2: Mapping of *heb3-1* and *heb7-1* mutations

(a,b) Map position of the *heb3-1* **(a)** and *heb7-1* **(b)** mutations. The markers used and the number of chromosomes at each marker position over the number of total chromosomes analyzed is shown. Scale bars, 20 kb.

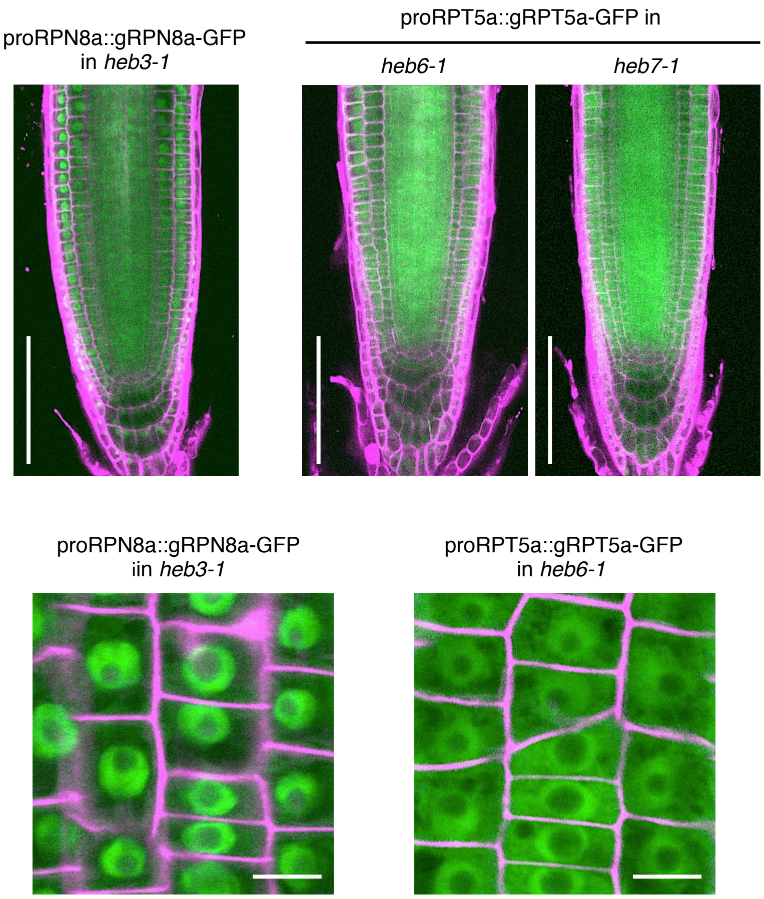

Supplementary Figure 3: GFP fused RPN8a and RPT5a expressions in RAM

Expression patterns of GFP fused RPN8a and RPT5a in RAM of transgenic plants used in Fig. 2c and 2d. Nine day-old seedlings grown on normal media were observed by confocal microscopy. Magnified images (lower panels) show subcellular localizations of each protein. Scale bars, 50 μm (upper) and 5 μm (lower).

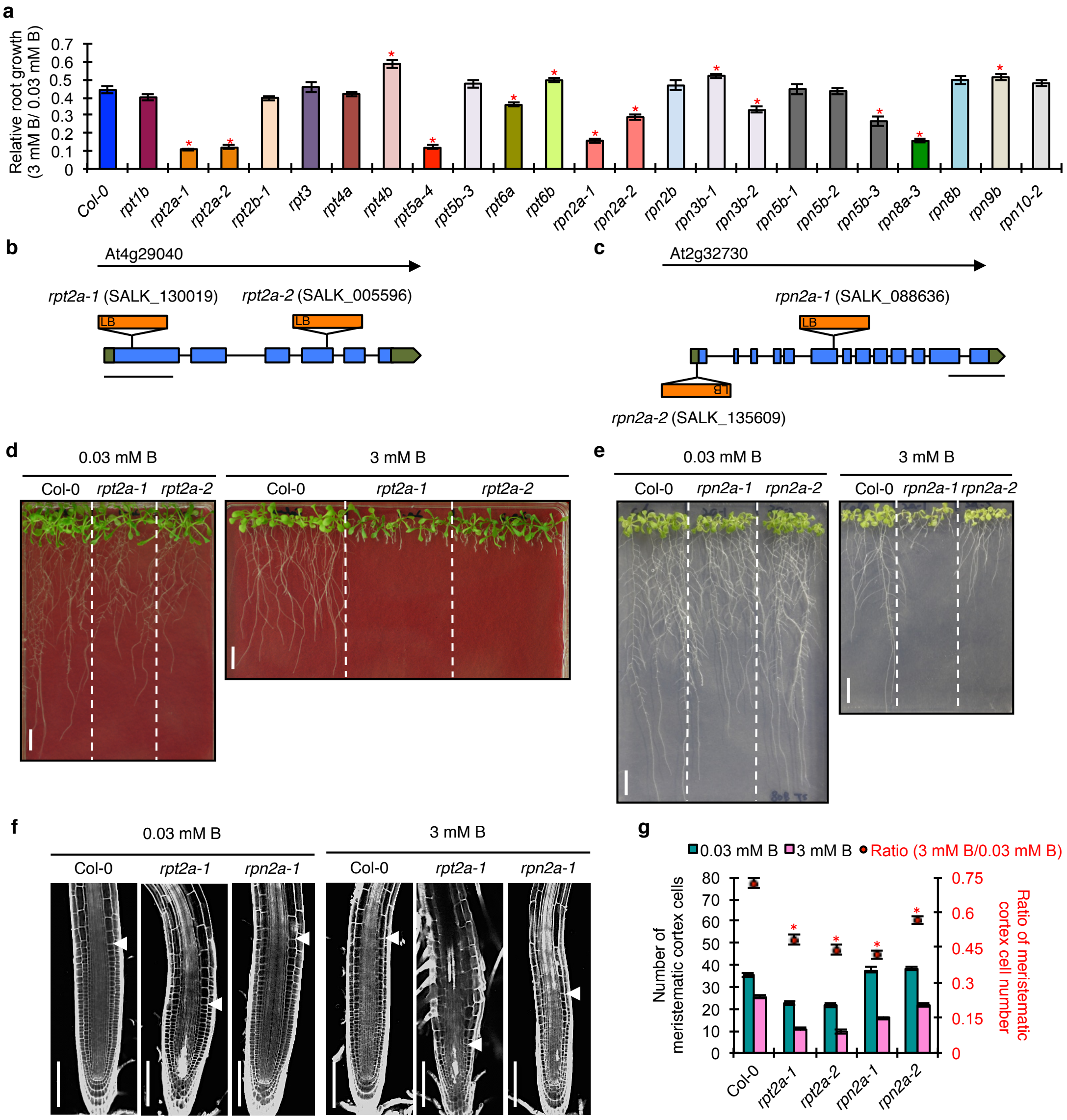

Supplementary Figure 4: RPT2a and RPN2a are also required for the tolerance to high-B stress in RAM

(a) Sensitivity of root growth of Col-0 and various mutants of RPs. Plants were grown on 0.03 and 3 mM B condition for 10 d. Then the primary root length was measured. Values were represented as ratio relative to the value under 0.03 mM B condition (n=10−20, average ± s.e.m; **P*<0.05, compared with Col-0, Student's *t*-test). **(b,c)** Gene structure and T-DNA insertion sites in *RPT2a* **(b)** and *RPN2a* **(c)**. Blue and green boxes indicate coding regions and untranslated

regions, respectively. **(d,e)** Short-root phenotypes of T-DNA inserted mutants of *RPT2a* **(d)** and *RPN2a* **(e)** grown under normal- (left) and high-B (right) conditions for 14 d. Scale bars, 1 cm. **(f)** Representative images of root morphology of the *rpt2a* and the *rpn2a* mutants under normal- and high-B conditions. Arrowheads indicate the border between meristem and elongating region. Scale bars, 50 μm. **(g)** Effect of high-B stress on the number of cortex cells in the RAM of Col-0, the *rpt2a,* and the *rpn2a* mutants. Red circles represent the ratio relative to the value under 0.03 mM B condition (n=10−16, average ± s.e.m; **P*<0.05, compared with Col-0, Student's *t*-test).

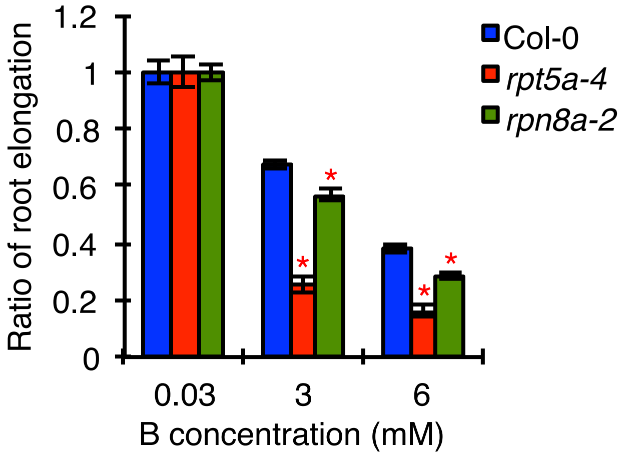

Supplementary Figure 5: RPT5a and PRN8a are required for post-embryonic tolerance to high-B stress

Sensitivity of root growth in Col-0, *rpt5a-4* and *rpn8a*-2 to high-B stress by the transplant method. Values are represented as ratios relative to the values at 0.03 mM B (n=14–16, average ± s.e.m; **P*<0.05, compared with the wild type, Student's *t*-test).

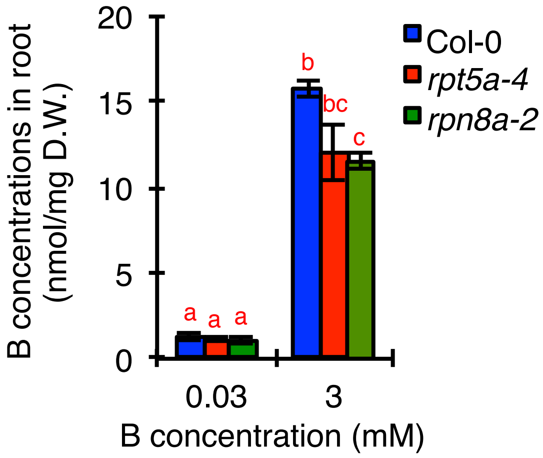

Supplementary Figure 6: Hypersensitivity of the RP subunit mutants is not caused by over accumulation of B

Boron concentrations in roots of Col-0, *rpt5a-4* and *rpn8a-2* grown under normal- and high-B conditions (n=4, average ± s.e.m; $P < 0.05$, one-way ANOVA and Tukey HSD).

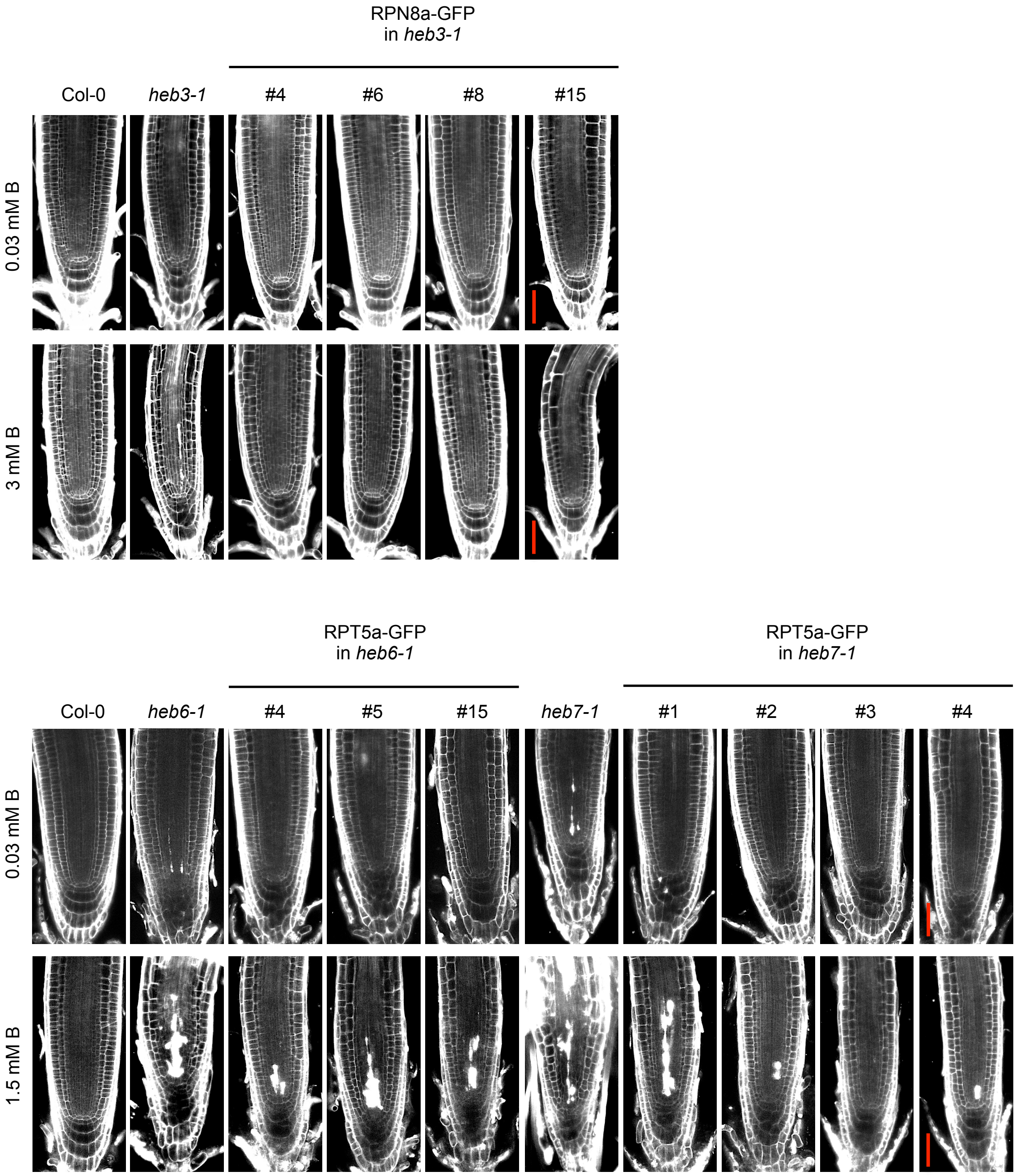

Supplementary Figure 7: RAM morphology of RPN8a-GFP expressing *heb3-1* and RPT5a-GFP expressing *heb6-1* and *heb7-1*

Representative images of root morphology of Col-0, *heb3-1*, *heb6-1*, *heb7-1*, RPN8a-GFP expressing *heb3-1*, and RPT5a-GFP expressing *heb6-1* and *heb7-1* transgenic plants grown under normal- and high-B conditions.

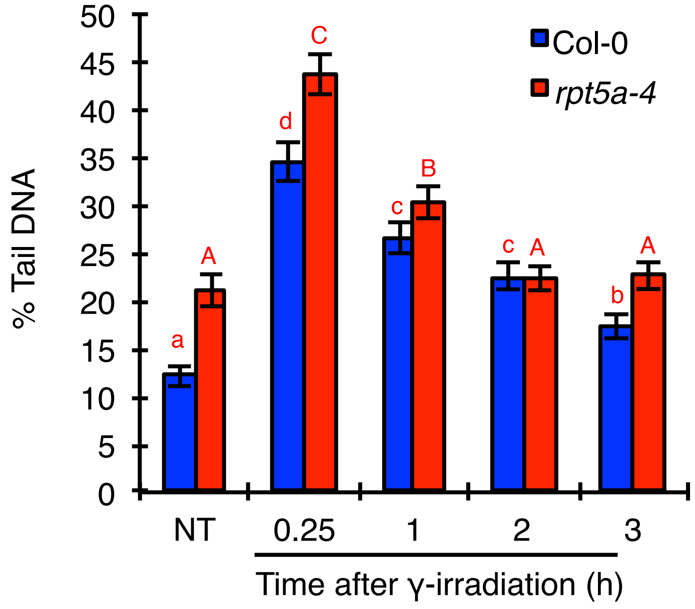

Supplementary Figure 8: Analysis of DSB repair in root tips of *rpt5a*

Time course of DSB levels in root tips of Col-0 and *rpt5a-4* after transient DSB induction by γ-irradiation (n=121, average ± s.e.m; P<0.05, one-way ANOVA and Tukey HSD). This data was used for the calculation of DSB repair kinetics shown in Fig. 4d.

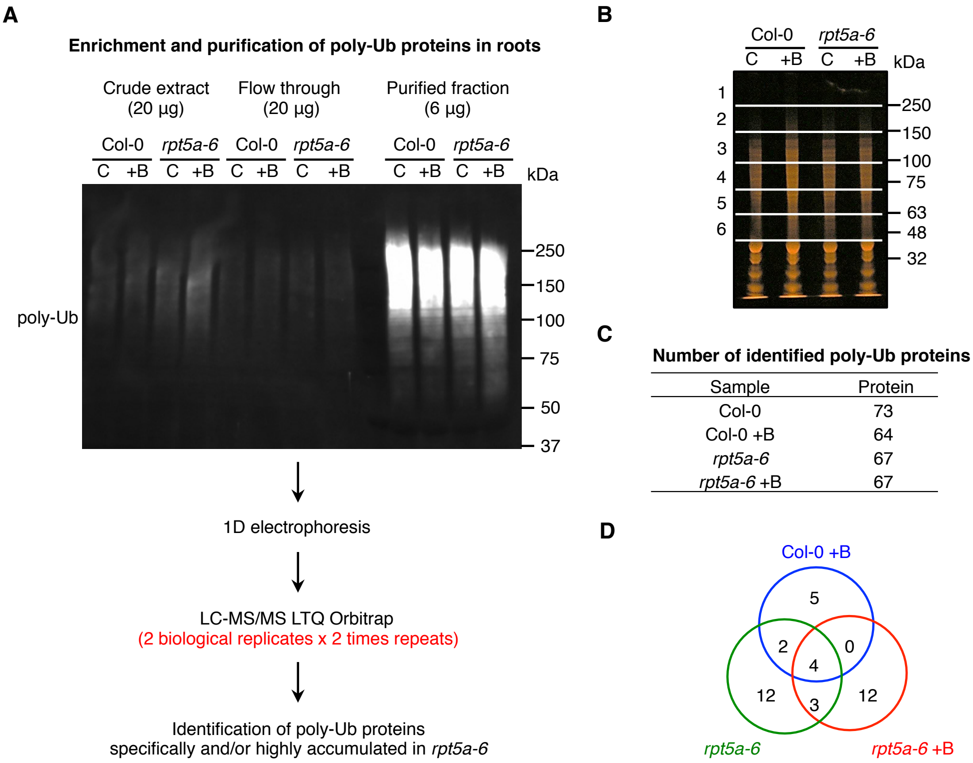

Supplemental Figure 9: Identification of poly-Ub proteins responsible for the hypersensitivity of the *rpt5a* mutants to high-B stress

(a) Schematic representation of identification of accumulated poly-Ub proteins in roots. Immunoblot analysis indicates enrichment of poly-Ub proteins in roots. **(b)** Proteins separated onto SDS-PAGE gel were divided into six fractions. Extracted proteins from each fraction were subjected to LC-MS/MS analysis. **(c)** Number of identified poly-Ub proteins in each sample. Among four independent analyses, only proteins detected at least two times were used. The details of the proteins were listed in Supplementary Data 1. **(d)** Comparison of identified poly-Ub proteins which accumulate depending on high-B stress, loss of RPT5a function, and both factors. Twelve proteins specifically accumulated in high-B treated *rpt5a-6* are listed in Supplementary Table 1.

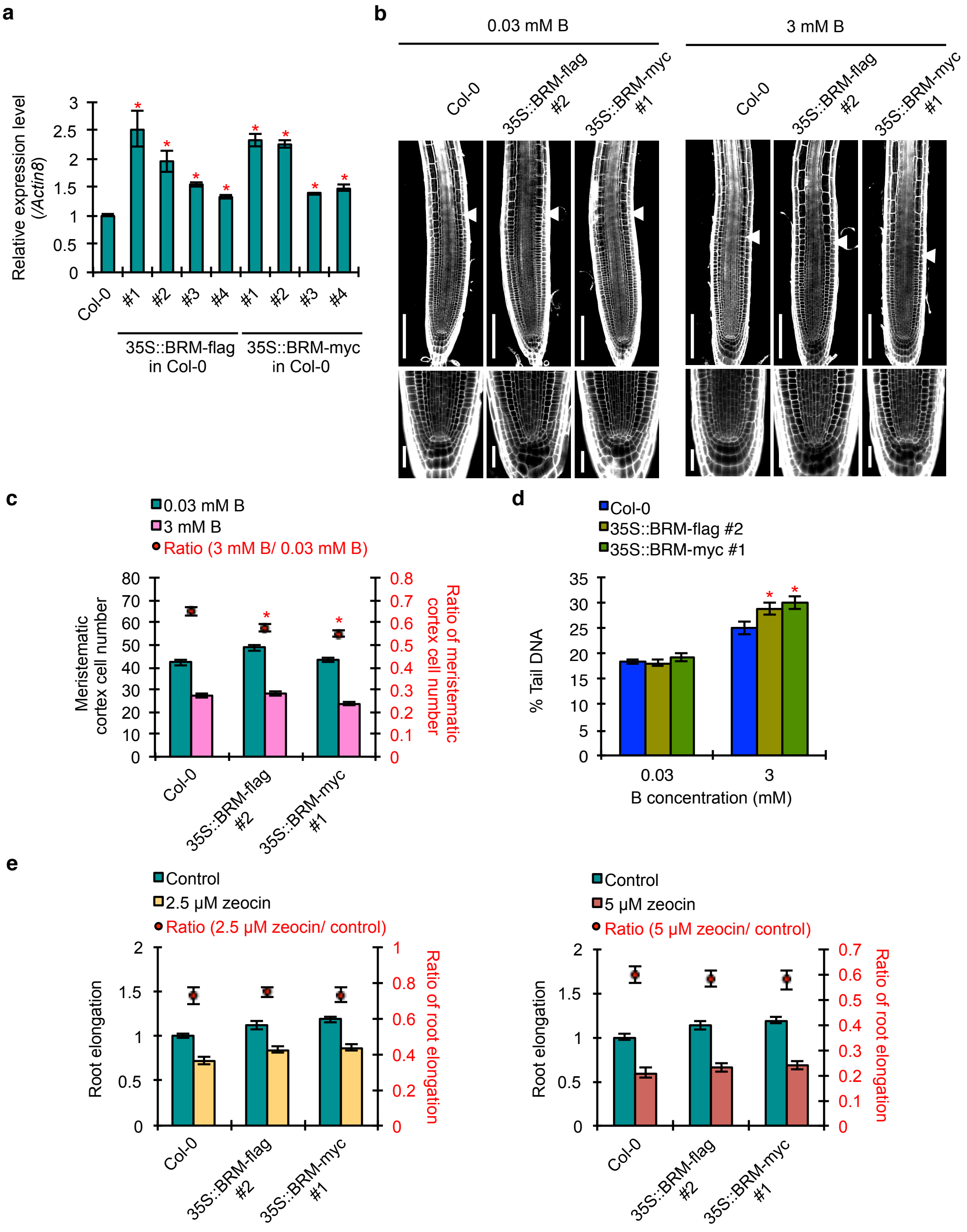

Supplementary Figure 10: Analysis of transgenic plants showing enhanced expression of BRM

(a) Expression levels of *BRM* mRNA in the roots of 7 d-old transgenic plants (n=4, average ± s.e.m; **P*<0.05, compared with Col-0, Student's *t*-test).

(b) Representative images of root morphology of two transgenic plants treated with normal- and high-B for 4 d. Arrowheads indicate the border between the meristem and elongating region. Below is the magnified image of stem cell niche. Scale bars, 50 μm.

(c) Effect of high-B stress on the number of cortex cells in the RAM of Col-0 and two transgenic plants. Red circles represent the ratio relative to the value under 0.03 mM B condition (n=19−20, average ± s.e.m; *P<0.05, compared with Col-0, Student's *t*-test).

(d) Levels of DSBs in root tips of two transgenic plants treated with normal- and high-B for 4 d (n=125 nuclei, average ± s.e.m; *P<0.05, compared with Col-0, Student's *t*-test).

(e) Sensitivity of root growth of two transgenic plants to DSBs-inducing factor, zeocin. Values for root elongation are represented as the ratio relative to the value of Col-0 under 0 μM zeocin condition. Red circles represent the ratio relative to the value under 0 μM zeocin condition (n=26, average ± s.e.m; *P<0.05, compared with Col-0, Student's *t*-test).

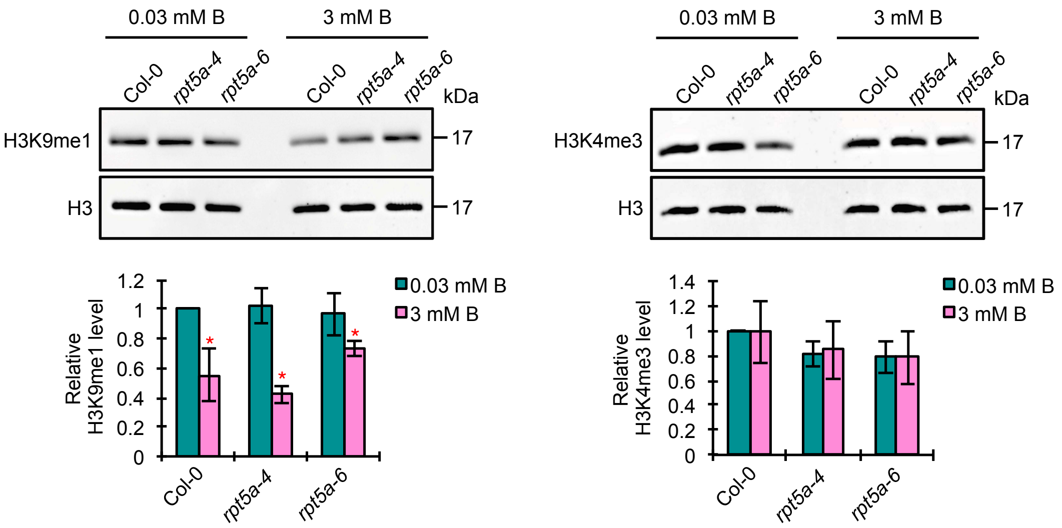

Supplementary Figure 11: Effects of high-B stress on histone modifications H3K9me1 and H3K4me3

Levels of histone H3 acetylation in roots of Col-0 and *the rpt5a* mutants grown under normal- and high-B conditions. Lower panel shows the relative level of indicated histone H3 modification normalized against total histone H3 (n=3, average ± s.e.m; **P*<0.05, compared with Col-0 under 0.03 mM B condition, Student's *t*-test).

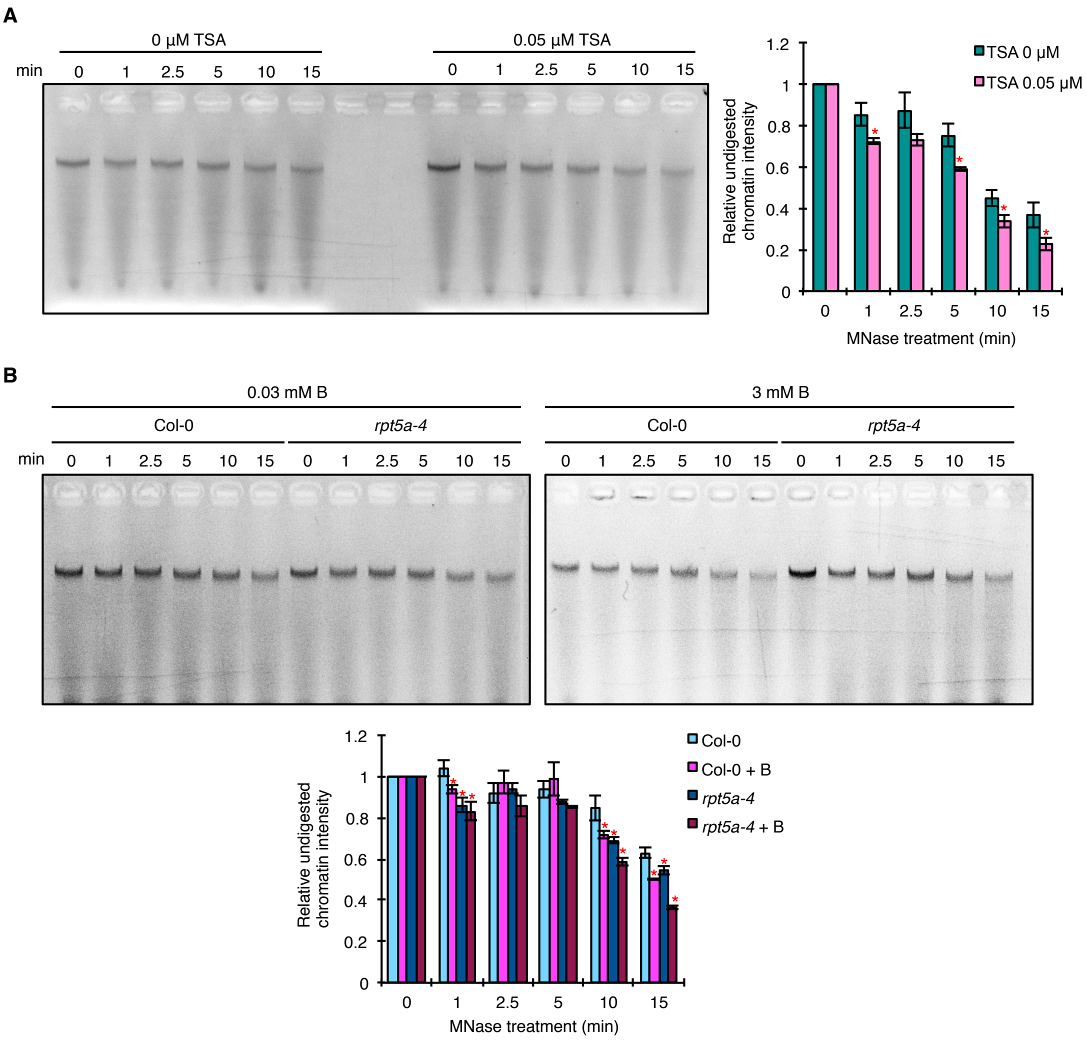

Supplementary Figure 12: High-B stress and TSA treatment reduces chromatin integrity

(a,b) Chromatin integrity was analyzed by MNase assay. After 5 d incubation on normal media, plants were transferred to media containing indicated concentration of TSA **(a)** or B **(b)**. Representative images of MNase assay were shown. Values were represented as ratio of undigested chromatin intensity relative to that under 0 min MNase treatment. Average ± s.e.m. (n=3) were shown. Asterisks indicate significant differences between Col-0 under the normal condition and treatment or mutation (*P*< 0.05, Student's *t*-test).

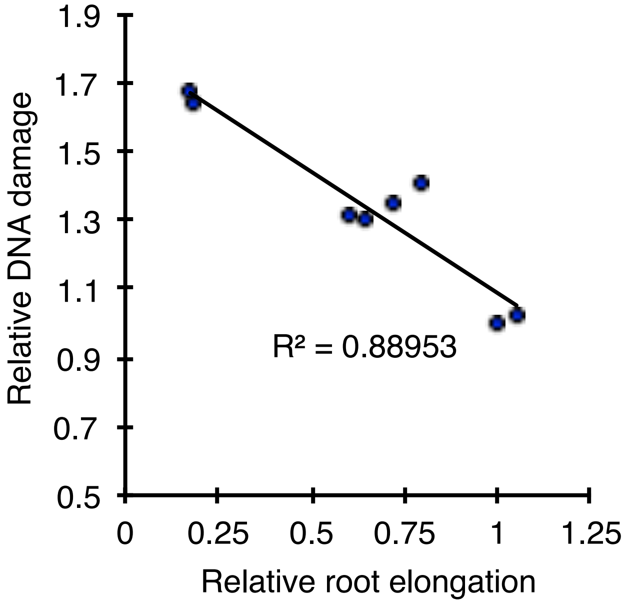

Supplementary Figure 13: DSBs in RAM is a major cause of root growth inhibition caused by high-B stress

Relationship between root growth and DSB levels in root tips under high-B conditions using data from Fig. 4b and Supplementary Fig. 5. A correlation between the extent of DSB levels and the extent of root growth inhibition was observed.

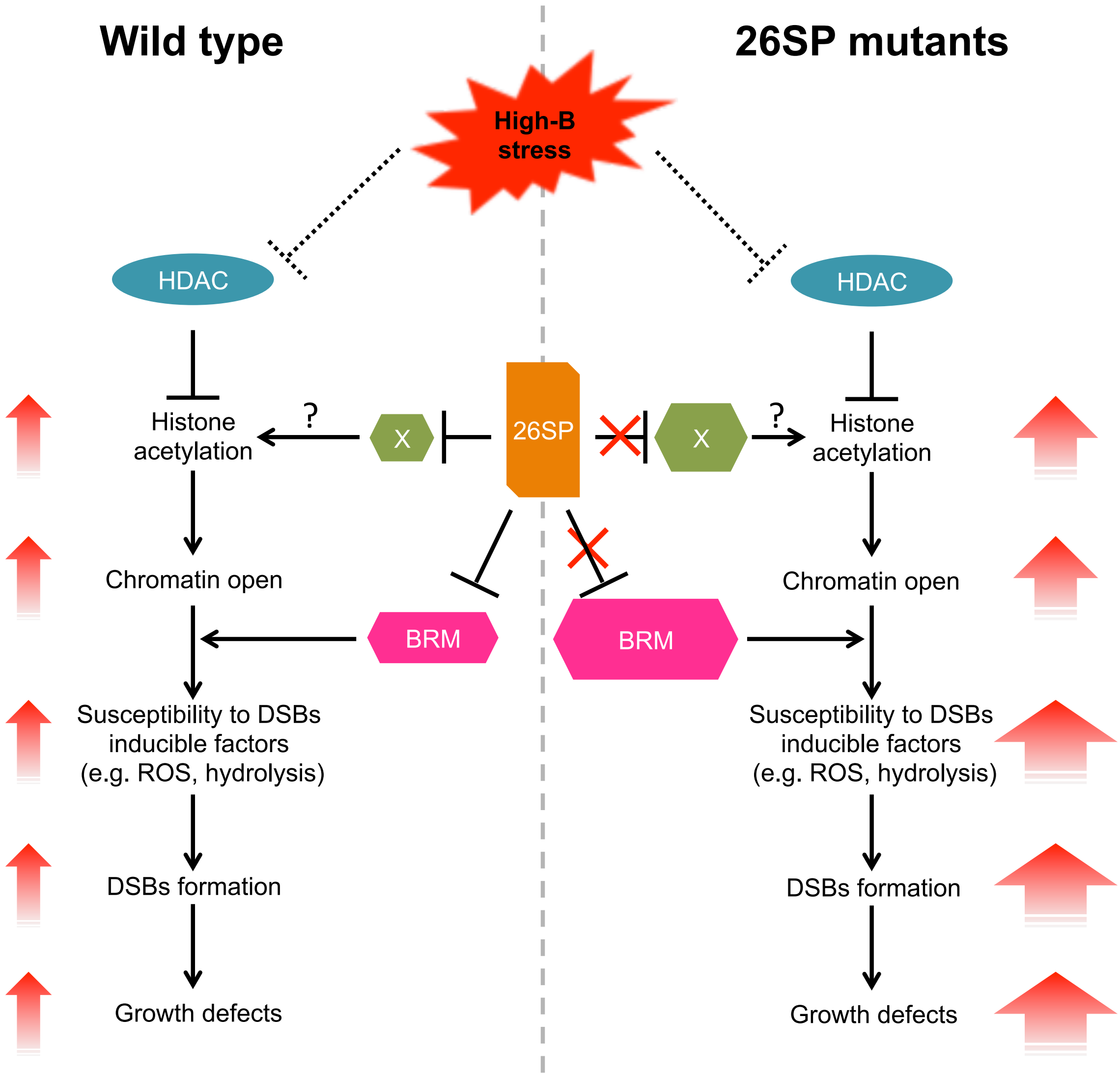

Supplementary Figure 14: A schematic model for the inhibitory mechanism of DSBs caused by high-B stress in *Arabidopsis*

High-B stress destabilizes chromatin *via* increased histone acetylation. 26SP degrades BRM, which preventing overfuncition of BRM on further chromatin destabilization and subsequent increased susceptibility to DNBs inducible factors. It is noted that the inhibitory effect of high-B stress on HDAC activities in *Arabidopsis* was not confirmed in this study.

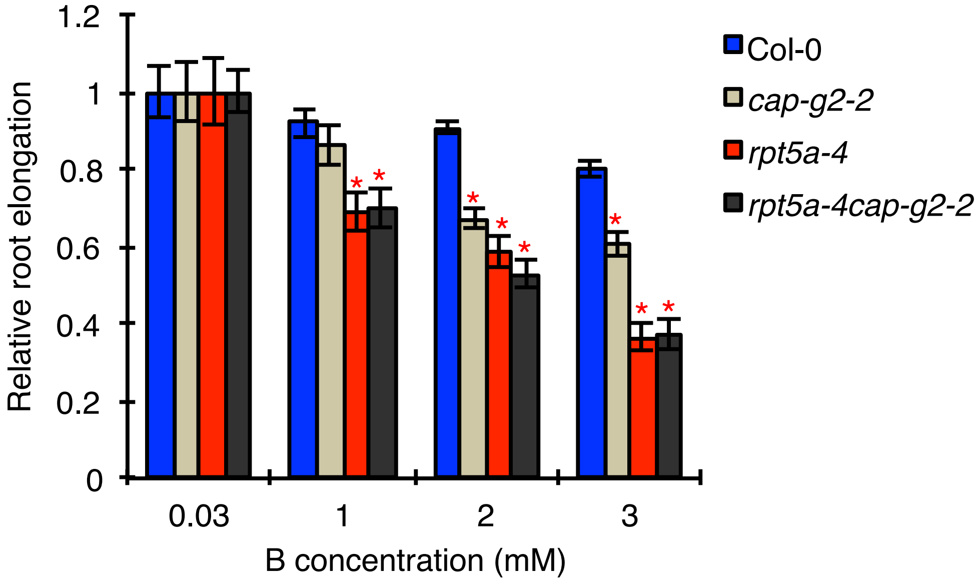

Supplementary Figure 15: 26SP and condensin II acts in the tolerance to high-B stress in a coordinated manner

Sensitivity of root growth of Col-0 and *rpt5a-4cap-g2-2* to high-B stress. After 5 d incubation on normal media, plants were transferred to media containing indicated concentrations of B. Then the elongated primary root length during additional 4 d incubation was measured. Values were represented as ratio relative to the value under 0.03 mM B condition. Average ± s.e.m. (n=14−19) were shown. Asterisks indicate significant differences between Col-0 and each mutant (P<0.05, Student's t-test). There are no significant differences between *rpt5a-4* and *rpt5a-4cap-g2-2* ($P \geqq 0.05$, Student's t-test).

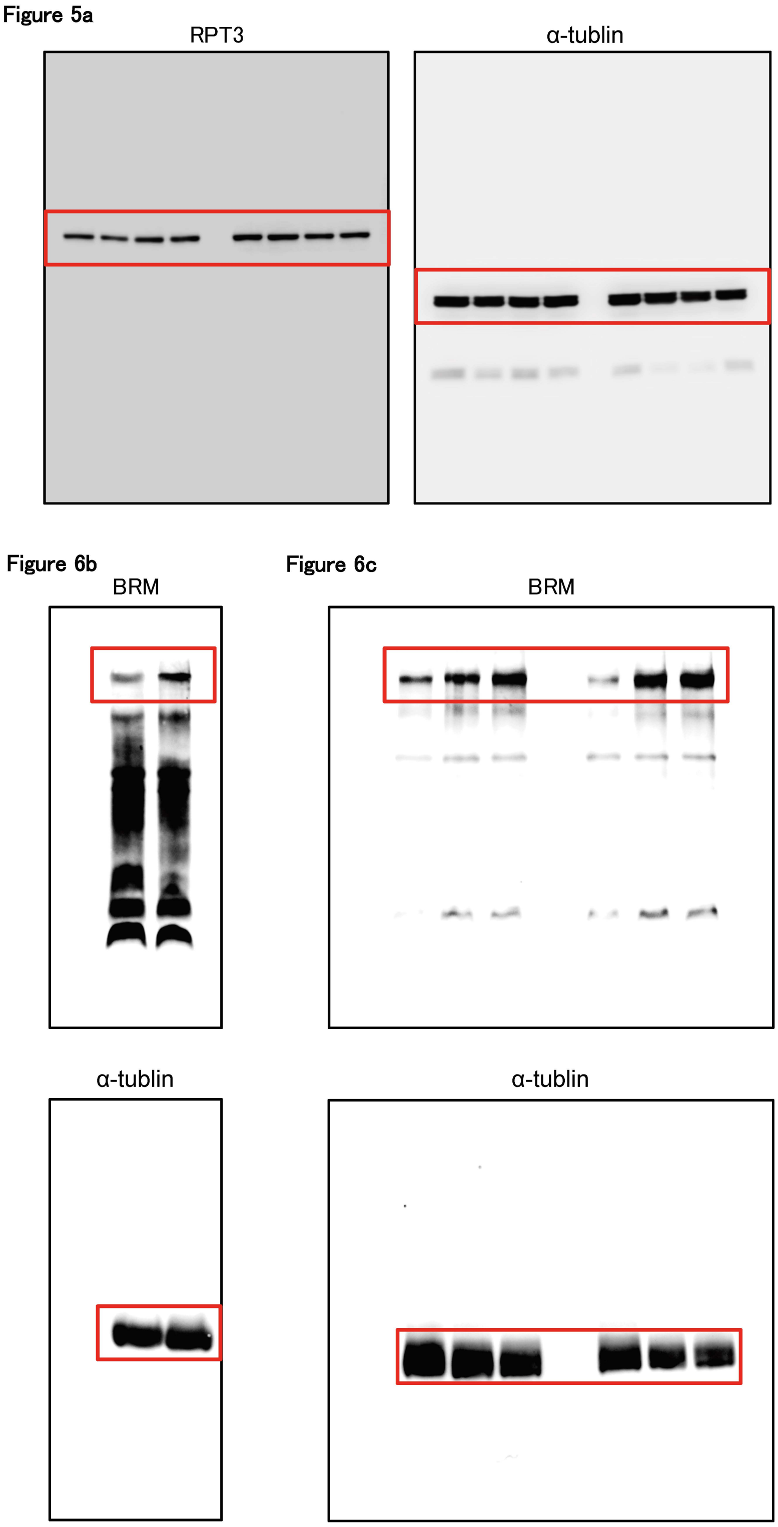

Supplementary Figure 16: Uncut blots

The red sections mark blot results shown in the indicated figures.

Figure 9a

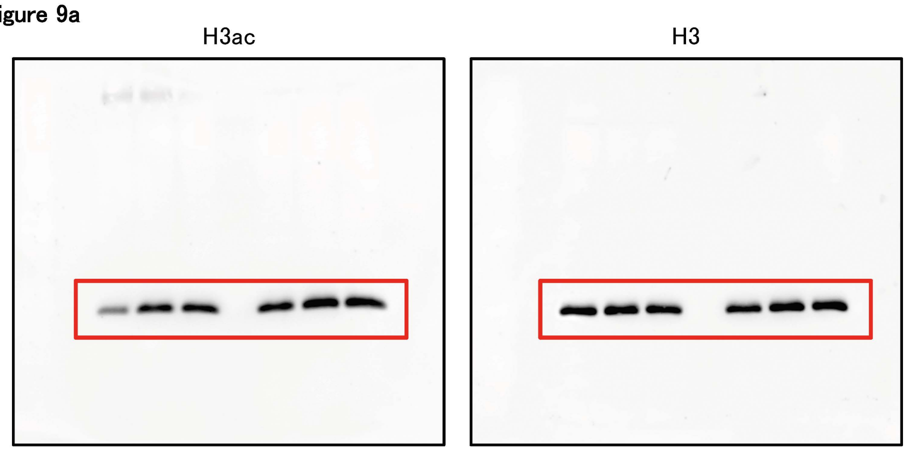

Figure 9g

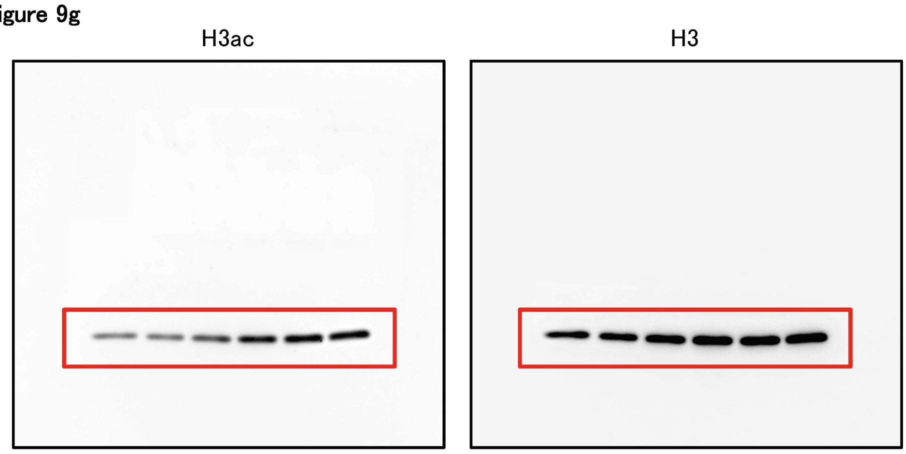

Figure 10e

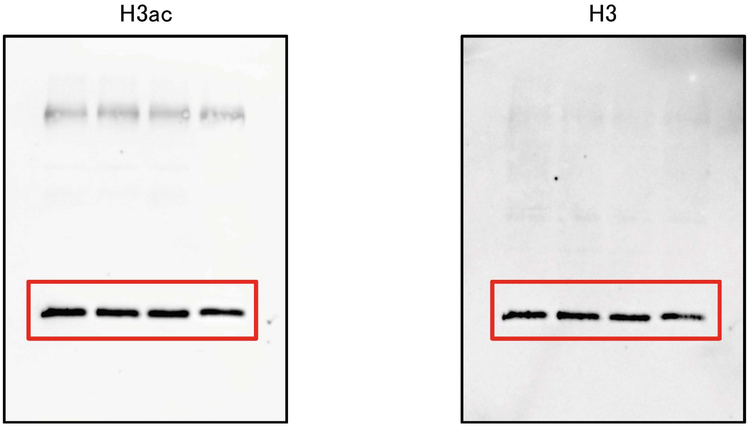

Supplementary Figure 17: Uncut blots

The red sections mark blot results shown in the indicated figures.

Supplementary Figure 11

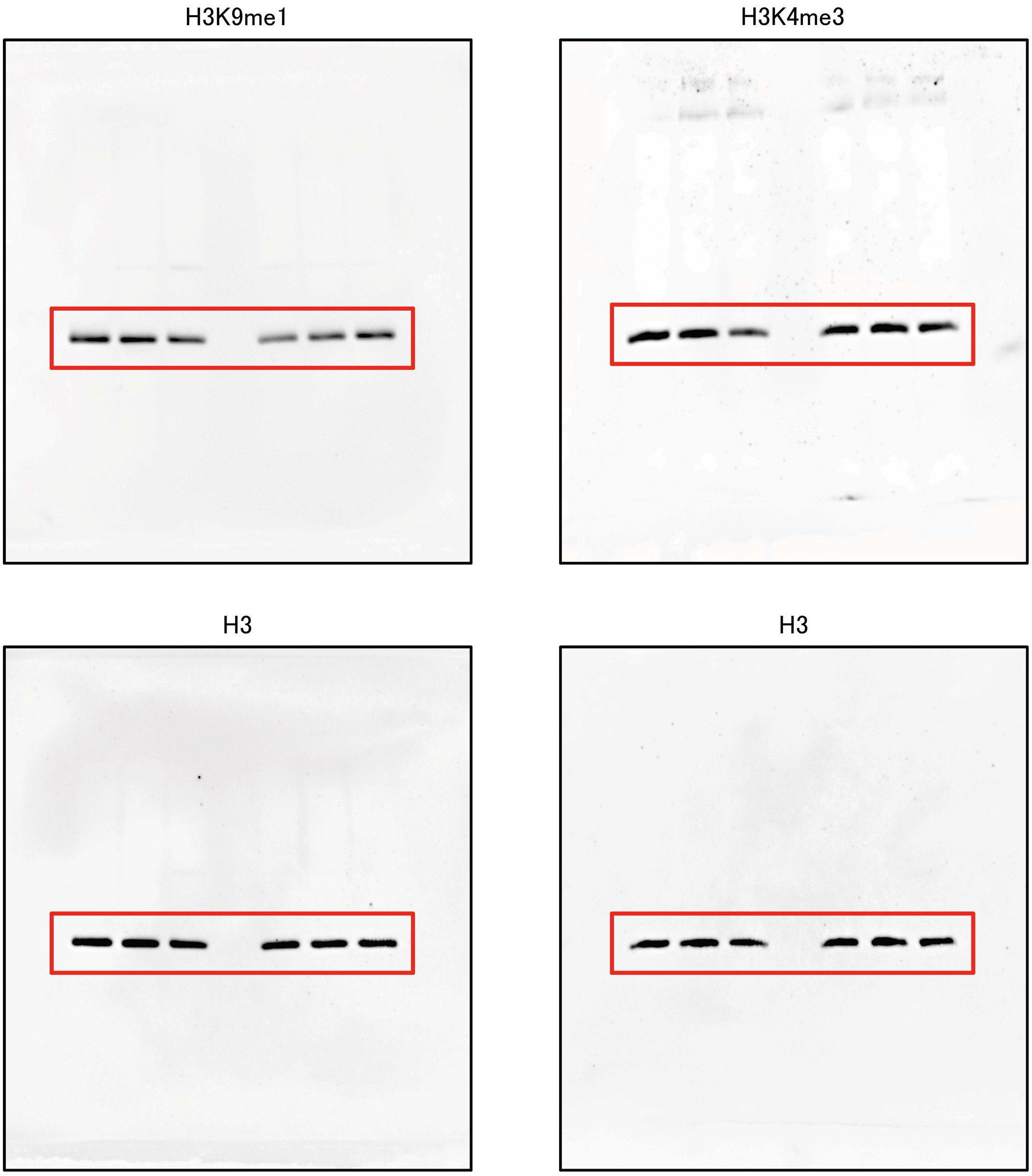

Supplementary Figure 18: Uncut blots

The red sections mark blot results shown in the indicated figures.

Supplementary Tables

Supplemenatry Table 1. Poly-Ub proteins specifically accumulated in *rpt5a-4* treated with high

AGI code	Annotation	Number of motifs		
		PEST	KEN-box	D-box
AT1G04940	TIC20 (Translocon at chroloplast membrane)	0	0	1
AT1G49340	PI4K (Phosphatidylinositol 4-kinase alpha)	2	0	9
AT1G59890	SNL5 (SIN3-like 5)	2	3	1
AT1G74430	MYB95	1	0	0
AT2G20805	DNA-binding storekeeper protein-related	0	0	0
AT2G46020	BRM/CHR2 (Chromatin remodeling 2)	2	1	2
AT3G06980	DEAD/DEAH box helicase	2	1	1
AT3G07980	MAPKKK6	3	1	3
AT3G58530	RNI-like superfamily	0	0	1
AT4G23950	Galactose-binding protein	0	1	0
AT5G10170	MIPS3 (Myo-inositol-3-phosphate synthase)	0	0	1
AT5G27220	Frigida-like protein	1	0	2

See also Supplementary Fig. 4 and Data 1 for the screening strategy and the list of detected proteins, respectively.

Supplementary Table 2. List of T-DNA inserted mutants used in this study

Gene		AGI code	Mutants
	RPT1a	At1g53750	
	RPT1b	At1g53780	SALK_106176 (*rpt1b*)
	RPT2a	At4g29040	SALK_130019 (*rpt2a-1*)
			SALK_005596 (*rpt2a-2*)
	RPT2b	At2g20140	SALK_043450 (*rpt2b-1*)
	RPT3	At5g58290	SALK019265 (*rpt3)*
RPTs	*RPT4a*	At5g43010	SALK_052732 (*rpt4a*)
	RPT4b	At1g45000	SALK_101982 (*rpt4b*)
	RPT5a	At3g05530	SALK_046321 (*rpn5a-4*)
			heb6-1/*rpt5a-5*
			heb6-2/*rpt5a-6*
	RPT5b	At1g09100	SAIL_293_H08 (*rpt5b-3*)
	RPT6a	At5g19990	SAIL_443_F04 (*rpt6a*)
	RPT6b	At5g20000	GABI_483G04 (*rpt6b*)
	RPN1a	At2g20580	
	RPN1b	At4g28470	
	RPN2a	At1g08140	SALK_088636 (*rpn2a-1*)
			SALK_135609 (*rpn2a-2*)
	RPN2b	At2g32730	SALK_024347 (*rpn2b*)
	RPN3a	At1g20200	
	RPN3b	At1g75990	SALK_117415 (*rpn3b-1*)
			SALK_088176 (*rpn3b-2*)
	RPN5a	At5g09900	
	RPN5b	At5g64760	SALK_134671 (*rpn5b-1*)
			SALK_133892 (*rpn5b-2*)
RPNs			SALK_127791 (*rpn5b-3*)
	RPN6	At1g29150	
	RPN7	At4g24820	
	RPN8a	At5g05780	SALK_151595 (*rpn8a-2)*
			heb3-1/*rpn8a-3*
	RPN8b	At3g11270	SALK_009871 (*rpn8b*)
	RPN9a	At5g45620	SALK_043310 (*rpn9b*)
	RPN9b	At4g19006	
	RPN10	At4g38630	SALK_009616 (*rpn10-2*)
	RPN11	At5g23540	
	RPN12a	At1g64520	
	RPN12b	At5g42040	
	BRM	At2g46020	SALK_088462 (*brm-3*)
	HDA6	AT5g63110	*axe1-4*
	HDA19	AT4g38130	SALK_139445

Supplementary Table 3. Primers used in this study

Experiment	Primer Name	Primer sequence (5' to 3')	Remarks
T-DNA insertion	SALK (pROK2) LBb1	GATGGCCCACTACGTGAACCAT	
	SALK (pROK2) LBa1	TGGTTCACGTAGTGGGCCATCG	
	SALK (pROK2) LBb1.3	ATTTTGCCGATTTCGGAAC	
	SALK_088636 (*rpn2a-1*)_F	TTCTCATTCCTTTGTCAACCG	with LBb1
	SALK_088636 (*rpn2a-1*)_R	AGCATGCATAATGGCATTAGC	
	SALK_135609 (*rpn2a-2*)_F	AATCGTGTTTGTTTGTTCATACAC	with LBb1
	SALK_135609 (*rpn2a-2*)_R	AGCTCAAACAATCCAAATCCC	
	SALK_024347 (*rpn2b*)_F	TGAAGGTTGTCCAGGTTTCAG	with LBb1
	SALK_024347 (*rpn2b*)_R	ACAAATTTTCCGCATCATGAC	
	SALK_117415 (*rpn3b-1*)_F	CTGCAAAAACATTGAAAAGGG	with LBb1
	SALK_117415 (*rpn3b-1*)_R	AGACTCCGACACAACGTGATC	
	SALK_088176 (*rpn3b-2*)_F	ACGAAACAAAACAAAATCCCC	with LBb1
	SALK_088176 (*rpn3b-2*)_R	TCGGCACAAAAGAAGAGAGAC	
	SALK_134671 (*rpn5b-1*)_F	GGCTCCGGCTGATATACCTAC	with LBb1
	SALK_134671 (*rpn5b-1*)_R	ACCGGGTTCTCAAGCTCTTAG	
	SALK_133892 (*rpn5b-2*)_F	AAACCCAGAACAATGGATTCC	with LBb1
	SALK_133892 (*rpn5b-2*)_R	GCCTGAAAGATTCTTTCCACC	
	SALK_127791 (*rpn5b-3*)_F	CCCTTCTGTAAAGGAAAACCC	with LBb1
	SALK_127791 (*rpn5b-3*)_R	GCCTGAAAGATTCTTTCCACC	
	SALK_151595 (*rpn8a-3*)_F	TTGCTCAGGGATGTGAAAGAC	with LBb1
	SALK_151595 (*rpn8a-3*)_R	GGCCTAAGCCTAACGAACTTG	
	SALK_009871 (*rpn8b*)_F	AGAAGCTTTGCGACTTAAGGC	with LBb1
	SALK_009871 (*rpn8b*)_R	TTTATGTTCGCACAGGGTTTC	
	SALK_043310 (*rpn9b*)_F	TTGGATCTTCCAACTTTCGTG	with LBb1
	SALK_043310 (*rpn9b*)_R	TTCGATCTTGTATCGGTTTGG	
	SALK_009616 (*rpn10-2*)_F	GTTGCTAGATCAGGCAATTGC	with LBb1
	SALK_009616 (*rpn10-2*)_R	AAAACTACAGCTCGCATGTGG	
	SALK_046321 (*rpn5a-4*) _F	TCCAACATAGTCCTCTGCAC	with LBb1.3
	SALK_046321 (*rpn5a-4*) _R	TGATCTTGACTCGCAACGGA	
	SALK_049790 (*cap-h2-2*)_F	GGTTGCAAATTCAAATGTTCG	with LBb1.3
	SALK_049790 (*cap-h2-2*)_R	TTCCAATGAGGTCACAAAAGG	
	*brm-3*_F	ACCTTCCTTGTCGATTCTCC	with LBa1
	*brm-3*_R	GAAACTGTCCATGATGTACC	
Complementation	*RPN8a*/*HEB3* genomic_F	CACCTTTCTAAGCCTCGCCGAGAC	
	RPN8a/*HEB3* genomic_R	GCTGGTGGCAGGTATGGCCACAGGCTTT	
	RPT5a/*HEB6* genomic_F	CACCCTCTAGAGGTTCCCAATTAG	
	RPT5a/*HEB6* genomic_R	GGCGTAGTAGTTCAAGCTTG	
Overexpression	*BRM3* CDS_F	CACCATGCAATCTGGAGGCAGTGG	
	BRM3 CDS_R	TAAATGGCTAGGCCGTCTTTTACC	
RT-PCR	*BRCA1* (At4g21070)_F	CCATGTATTTTGCAATGCGTG	
	BRCA1 (At4g21070)_R	TGTGGAGCACCTCGAATCTCT	
	RAD51 (At5g20850)_F	CGAGGAAGGATCTCTTGCAG	
	RAD51 (At5g20850)_R	GCACTAGTGAACCCCAGAGG	
	GR1 (At3g52115)_F	GAAGGAGCAGACAAAGTGAG	
	GR1 (At3g52115)_R	GGTGAGATGGAAGTGATAGG	
	PARP1 (At4g02390)_F	GCTTTGGGAGACATGAATGAAC	
	PARP1 (At4g02390)_R	AAGTGGAACAACAACACCGTCT	
	Actin8 (At1g49240)_F	GCCAGATCTTCATCGTCGTG	
	Actin8 (At1g49240)_R	TCTCCAGCGAATCCAACCTT	

Supplementary Table 4. Primers for mapping of *heb m*utations

Loci	Marker name	Type	Primer Sequence (5' to 3')	Product size	
				Col-0	Ler
heb3	T32M21	SSLP	GAGAACCAGATGATCCAAGTCC<br>ATCCACCAATGCTACGTTCC	193	183
	NHFD	SSLP	AAAAACCCAAACTTTCTATTTATAC<br>ACTTCGCTTCAAGTAAAGAGG	124	113
	F8L15	SSLP	AAAAATCAGCATTGTTGTGGTT<br>GCTTGACTCCGGTGTTGACT	240	196
	K18I23	SSLP	TTGCTACAACTTGCGATTCC<br>TTAAAGGTTTCCGGTGTACG	213	223
	MOP10	SSLP	CTTGCACTACATGTCCATAGAACC<br>ATCGTGAGCCTTATCAACTTGC	248	259
	MJJ3a	SSLP	TGCCAAAAGACAGAAACGAA<br>ATTGATTTGCAAAATGAGTATCC	145	112
	MJJ3b	SSLP	GGATCCAGGGTCTGACTCAA<br>GAACCAGCAGCAAGTGAACA	177	168
	MJJ3c	SSLP	CCTCTCCCTTGGTTCTTTCTTC<br>CTGACTTCAGCTTTGGGTAAGG	222	234
heb6	nga172	SSLP	CATCCGAATGCCATTGTTC<br>AGCTGCTTCCTTATAGCGTCC	162	136
	T12H1	SSLP	AGGTTAGCGATTGAAGTTTCG<br>AAAAGCAGTGTTGGGGAAGA	246	233
	F22F7a	SSLP	TTGTCACCACATTAATTCCAAG<br>GAACCCCTGAACTCTGCAAC	242	177
	F22F7b	SSLP	CAATGCCAGCTGCAAAGTTA<br>CGCCGTTAGTTTACCCAAAA	220	203
	F18C1a	SSLP	ACTGCTGCTCCTAACCAGTCTC<br>GCAGTTGAACAATACCCTCCTC	210	200
	F18C1b	SSLP	CCGGTGGAGAAAAGAACAAA<br>GGAAATCCGGCTAGTGAGAA	165	161
	F18C1c	SSLP	TTTGTGCTGGGTTTCAATCA<br>TGTGTGATGGAATCAATTTGG	157	149
