# Supplementary material for: Proteasomal degradation of BRAHMA promotes Boron tolerance in Arabidopsis
Source: Nat Commun. 2018 Dec 11;9:5285. doi: 10.1038/s41467-018-07393-6 (PMC6290004; doi:10.1038/s41467-018-07393-6)
Supplement: Supplementary file 3 — Description of Additional Supplementary Files [file 41467_2018_7393_MOESM3_ESM.pdf]

Description of Additional Supplementary Files:

File Name: Supplementary Data 1

Description: The list of poly-Ub proteins identified in the roots of wild type and *rpt5a-4* under normal and high-B conditions.
